# Validation of the nearest-neighbor model for Watson–Crick self-complementary DNA duplexes in molecular crowding condition

**DOI:** 10.1093/nar/gkz071

**Published:** 2019-02-08

**Authors:** Saptarshi Ghosh, Shuntaro Takahashi, Tamaki Endoh, Hisae Tateishi-Karimata, Soumitra Hazra, Naoki Sugimoto

**Affiliations:** 1 Frontier Institute for Biomolecular Engineering Research (FIBER), Konan University, 7-1-20 Minatojima-Minamimachi, Chuo-ku, Kobe, 650-0047, Japan; 2 Graduate School of Frontiers of Innovative Research in Science and Technology (FIRST), Konan University, 7-1-20 Minatojima-Minamimachi, Chuo-ku, Kobe, 650-0047, Japan

## Abstract

Recent advancement in nucleic acid techniques inside cells demands the knowledge of the stability of nucleic acid structures in molecular crowding. The nearest-neighbor model has been successfully used to predict thermodynamic parameters for the formation of nucleic acid duplexes, with significant accuracy in a dilute solution. However, knowledge about the applicability of the model in molecular crowding is still limited. To determine and predict the stabilities of DNA duplexes in a cell-like crowded environment, we systematically investigated the validity of the nearest-neighbor model for Watson–Crick self-complementary DNA duplexes in molecular crowding. The thermodynamic parameters for the duplex formation were measured in the presence of 40 wt% poly(ethylene glycol)200 for different self-complementary DNA oligonucleotides consisting of identical nearest-neighbors in a physiological buffer containing 0.1 M NaCl. The thermodynamic parameters as well as the melting temperatures (*T*_m_) obtained from the UV melting studies revealed similar values for the oligonucleotides having identical nearest-neighbors, suggesting the validity of the nearest-neighbor model in the crowding condition. Linear relationships between the measured Δ*G*°_37_ and *T*_m_ in crowding condition and those predicted in dilute solutions allowed us to predict Δ*G*°_37_, *T*_m_ and nearest-neighbor parameters in molecular crowding using existing parameters in the dilute condition, which provides useful information about the thermostability of the self-complementary DNA duplexes in molecular crowding.

## INTRODUCTION

Estimation of the stability of nucleic acid duplexes is important in various molecular biology techniques, such as polymerase chain reaction (PCR) (1), hybridization-induced sequencing (2) and antigene targeting in gene therapy (3). Furthermore, the recent gene editing technique using the CRISPR/Cas system is also based on the hybridization of a nucleic acid duplex (4). Knowledge of sequence dependence of the thermodynamic parameters is also important to understand the key biological processes, such as DNA replication, transcription, mutation and repair (5–7). Therefore, estimation of the thermostability of nucleic acids is always one of the intriguing aspects of nucleic acid research.

The nearest-neighbor model developed by Tinoco *et al.* is one of the extensively used methods to predict the thermostability of Watson–Crick nucleic acid duplexes, assuming a two-state melting behavior of the duplexes (8,9). According to this model, the thermodynamic values (Δ*H*°, Δ*S*° and Δ*G*°_37_) for a duplex formation consists of three terms: (i) a free energy change for helix initiation to form a first base pair in the double helix, (ii) a free energy change for helix propagation as the sum of each subsequent base pair and (iii) a free energy change of mixing entropy term for self-complementary strands. Based on this model, the nearest-neighbor parameters were developed by some groups, including us, and have been commonly used to predict the stabilities of different types of DNA–DNA, RNA–RNA, RNA–DNA duplexes, and duplexes formed by peptide nucleic acid (PNA) and DNA (10–18).

At present, the nearest-neighbor parameters have been used in software packages for secondary structure prediction, siRNA design, non-coding RNA detection, DNA primer design and nanostructure design (19–25). Considering the importance of the model in both experimental technique and theoretical modeling, the parameters of the nearest-neighbor model are being improved by modifying the methodology as well as analysis procedure (26–28). However, all predictions using the nearest-neighbor model assumed the nucleic acid under dilute solutions, which do not reflect the actual environment of the cell. Thus, improvement of the nearest-neighbor parameters for prediction of the nucleic acid thermostabilities under intracellular conditions is the key to technological development, based on the nucleic acid chemistry within cells. The most radical difference between dilute solutions and the intracellular environment is the presence of high concentrations of macromolecules (200–400 mg/ml) in cells that eventually occupy up to 40% of a cell’s volume (29). The complex composition inside the cell restricts physicochemical studies; thus, a few studies are available on the stabilities of nucleic acid structures inside the cell (30,31). To estimate the behavior of nucleic acids of interest in cells, their physicochemical properties have been investigated by mimicking the intracellular environment using large amounts of cosolutes, which are inert to nucleic acids (32–40). Many of these studies demonstrated considerably large differences when comparing nucleic acid behavior in the presence and absence of cosolutes. Thus, improved nearest-neighbor parameters for the prediction of stabilities of the oligonucleotides in cells are of great interest, but the validity of the nearest-neighbor model in the molecular crowding condition has not been established. In one of our earlier works, while determining the role of hydration for the stabilities of nucleic acid structures in the presence of cosolutes, we found that DNA duplexes consisting of the same nearest-neighbor composition have similar stabilities in the presence of cosolutes (39). This observation indicates that the nearest-neighbor model may also be applicable in the molecular crowding condition. Therefore, a systematic study is required for establishment of the validity of this model in molecular crowding.

In this work, we investigated the applicability of the nearest-neighbor model for Watson–Crick self-complementary DNA duplexes in molecular crowding condition induced by poly(ethylene glycol) having average molecular weight 200 (PEG 200). Self-complementary genomic DNAs gained notable importance in gene therapy as promising delivery vectors due to their high transduction efficiency (41). Therefore, knowledge of the stabilities of the self-complementary DNA duplexes in a cell-mimicking crowding environment would be of great importance for designing antisense oligonucleotides. We performed UV melting studies for nine pairs of self-complementary DNA sequences (sequences 1–9 in Table 1) having identical nearest-neighbors in the presence of 40 wt% PEG 200 in a physiological buffer condition. As a result, the analyzed thermodynamic parameters (Δ*H*°, Δ*S*° and Δ*G*°_37_) for the duplex formation as well as melting temperatures (*T*_m_) for the sequences in each pair exhibited similar values, which suggest that the nearest-neighbor model is valid even in a molecular crowding condition. Experiments also revealed similar values of Δ*H*°, Δ*S*°, Δ*G*°_37_ and *T_m_* for the sequences with identical nearest-neighbors in the presence of two different cosolutes: dextran 70 and Ficoll 70. Both cosolutes have an average molecular weight of 70 000, which differs largely from that of PEG 200. Moreover, dextran 70 and Ficoll 70 exhibit different physicochemical properties from PEG 200. The results with the cosolutes confirmed the validity of the model. Furthermore, we established the linear free-energy relationship between the measured values in the crowding conditions and predicted values in the dilute condition using existing nearest-neighbor parameters. The predicted values for oligonucleotides in 0.1 M NaCl were calculated from their standard values in 1 M NaCl using the linear equations reported by our group (42). Based on our proposed linear relation, we successfully calculated the nearest-neighbor parameters of Δ*G*°_37_ in crowding condition by a numerical approach. This study allows the prediction of the DNA duplex stabilities in cell-like crowded environments, which will be beneficial for important molecular biology techniques.

**Table 1.** Self-complementary DNA sequences with nearest-neighbor frequencies^a^

		Nearest-neighbor bases set present in duplex									
No.	Sequence	dAA dTT	dAT dTA	dTA dAT	dCA dGT	dGT dCA	dCT dGA	dGA dCT	dCG dGC	dGC dCG	dGG dCC
1a	d(CCGCGG)								2	1	2
1b	d(CGGCCG)^b^								2	1	2
2a	d(GGACGTCC)^b^					2		2	1		2
2b	d(GACCGGTC)					2		2	1		2
3a	d(CGTCGACG)^c^					2		2	3		
3b	d(CGACGTCG)					2		2	3		
4a	d(CAAGCTTG)^b^	2			2		2			1	
4b	d(CTTGCAAG)	2			2		2			1	
5a	d(CGGTACCG)			1		2			2		2
5b	d(CCGTACGG)			1		2			2		2
6a	d(GATCCGGATC)		2					4	1		2
6b	d(GGATCGATCC)		2					4	1		2
7a	d(ATGAGCTCAT)^b^		2		2		2	2		1	
7b	d(ATCAGCTGAT)		2		2		2	2		1	
8a	d(TGCCGCGGCA)				2				2	3	2
8b	d(TGGCGCGCCA)				2				2	3	2
9a	d(CATAGGCCTATG)		2	2	2		2			1	2
9b	d(CTATGGCCATAG)		2	2	2		2			1	2
10	d(AGTCATGACT)		1		2	2	2	2			
11	d(GCGAATTCGC)^c^	2	1					2	2	2	
12	d(ATCGCTAGCGAT)		2	1			2	2	2	2	
13	d(GACGACGTCGTC)					4		4	3		
14	d(GCAAGCCGGCTTGC)^d^	2			2		2		1	4	2
15	d(CGATCGGCCGATCG)		2					4	4	1	2
16	d(CATATGGCCATATG)^b^		4	2	4					1	2
17	d(CAAGATCGATCTTG)	2	2		2		2	4	1		
18	d(CGCGTACGCGTACGCG)^c^			2		4			6	3	
19	d(CGCAAGCCGGCTTGCG)^d^	2			2		2		3	4	2

^a^Oligonucleotides are reported by SantaLucia *et al.* (14)^b^ and us (13^c^,^44^^d^).

## MATERIALS AND METHODS

### Materials

All the synthetic DNA oligonucleotides used in this work, listed in Table 1, were purchased from Japan Bio Services Co and purified using high-performance liquid chromatography (HPLC). DNA samples were dissolved in Milli-Q water and stocked in −20°C until use. The concentrations of the single-stranded oligonucleotides were determined by measuring the absorbance at 260 nm at 90°C using the extinction coefficients. Poly(ethylene glycol) 200 (Wako Pure Chemical Industries, Japan), dextran 70 (TCI, Japan) and Ficoll 70 (GE healthcare, Sweden) were used as cosolutes without further purification. Disodium hydrogen phosphate (Na_2_HPO_4_) and sodium chloride (NaCl) were purchased from Wako Pure Chemical Industries (Japan), and disodium ethylenediaminetetraacetate (Na_2_EDTA) was purchased from Dojindo Molecular Technologies (Japan) and all these chemicals were used as received.

### UV melting measurement

Absorption spectra were measured on a Shimadzu 1800 spectrophotometer with a thermoprogrammer. All the experiments were conducted in a buffer containing 0.1 M NaCl, 10 mM Na_2_HPO_4_ and 1 mM Na_2_EDTA in the presence of cosolutes with specific weight percentages. We adjusted the pH of the buffer to 7.0 after adding the cosolutes (PEG 200, dextran 70 and Ficoll 70) to maintain the pH of the buffer solution. For melting experiments, concentrations of oligonucleotides were varied over a 50–100 fold range. The DNA solutions were kept at 90°C for 5 min, followed by the decrease of temperature from 90 to 0°C at a rate of 1°C min^−1^ to anneal the duplexes. Thereafter, the samples were heated from 0 to 90°C at a rate of 0.5°C min^−1^ to melt the duplex after keeping them at 0°C for 5 min. Condensation of water on the cuvette exterior at low temperature was avoided by flushing with a constant stream of dry N_2_ gas.

### Determination of thermodynamics for duplex formation

Thermodynamic parameters (Δ*H*°, Δ*S*° and Δ*G*°_37_) for self-complementary DNA duplexes were determined from the *T_m_*^−1^ versus ln (*C*_t_) plots as we described in our earlier study (43). To systematically obtain the *T*_m_ values, we developed a Microsoft Excel spreadsheet embedded with the calculation system for all the thermodynamic parameters (see Supplementary Data and Supplementary Figure S1). Briefly, this spreadsheet analyzes the linear baselines in the region of lower and upper temperatures of the melting profiles. The temperature ranges and the number of measurement points to be considered for the baselines were set arbitrarily according to the obtained data. Then, the median between upper and lower baselines is drawn to find the intersection with the melting profile. The temperature at the intersection is *T*_m_. After collecting 10–12 individual data from different series of DNA concentrations, we determined the *T*_m_^−1^ versus ln (*C*_t_) plots. From the slope and intercept of the linear plots, thermodynamic parameters were calculated using the following equations:

(1) \begin{equation*}{T_{\rm m}}^{ - 1} = R\,{\rm{ln}}\,\left( {{C_{\rm{t}}}} \right)/\Delta H^\circ + \Delta S^\circ /\Delta H^\circ \end{equation*}

(2) \begin{equation*}\Delta G{^\circ _{37}} = \Delta H^\circ -310.15 \bullet \Delta S^\circ \end{equation*}

where *R* is the gas constant and *C*_t_ is the total strand concentration of the oligonucleotides. For calculation of the parameters using equation (1), we assumed the difference in heat capacities (Δ*C*_p_) of the two states (single strand and duplex) was zero, as is standard practice (10–18). Zero Δ*C*_p_ presumed that Δ*H*° and Δ*S*° are temperature-independent in the experimental temperature range. Folding of nucleic acids is often associated with a finite value of Δ*C*_p_ (44), and neglecting Δ*C*_p_ may affect the precise values of the thermodynamic parameters. However, temperature-dependent changes in Δ*H*° and Δ*S*° largely offset one another in Δ*G*°(44). Compared with Δ*H*° and Δ*S*°, Δ*G*° and *T*_m_ values are relatively insensitive to the Δ*C*_p_ change (15), although large Δ*C*_p_ values have been specifically observed for oligomers with dangling ends (45). Since in the crowding condition, *T*_m_ values for most of the studied sequences were not far from the physiological temperature (37°C), the zero Δ*C*_p_ approximation should be adequate for stability estimation of these DNA duplexes at 37°C due to minimal extrapolations.

### Circular dichroism (CD) measurements

Circular dichroism (CD) spectra were obtained on a JASCO J-1500 spectropolarimeter equipped with a temperature controller. The experimental temperature was 4°C. The cuvette-holding chamber was flushed with a constant stream of dry N_2_ gas to avoid water condensation on the cuvette exterior. The CD spectra were measured from 200 to 340 nm in 0.1 cm path-length cuvettes with a scan rate of 50 nm min^−1^. The concentration of the samples was 20 μM in a buffer containing 0.1 M NaCl, 10 mM Na_2_HPO_4_ (pH 7.0) and 1 mM Na_2_EDTA with or without 40 wt% PEG 200.

## RESULTS

### Choice of sequences

The sequences shown in Table 1 were designed or selected from the literature where they exhibited two-state melting behavior. It was shown that the *T*_m_s of short duplexes of Watson–Crick DNA decrease in the presence of PEG 200 (36,38). Therefore, we chose those sequences with sufficiently high *T*_m_s (over 35°C) in dilute condition so that even in the crowding condition we can measure the *T*_m_s precisely. The sequences have different combinations of nearest-neighbor frequencies covering all 10 nearest-neighbor sets for DNA duplex formation. In addition to the combinations of the nearest-neighbor sets, the formation of the first base pairs in the double helix also affects the stability of the duplex. It is known as ‘initiation factor’ and achieved by either A•T or G•C base pairs (9). Since it is possible that the initiation factor also affects the stability of the duplex in the crowding condition, we chose a few sequences having A•T initiation and others with G•C initiation to include both the initiation factors in our designed sequences. It is reported that in dilute condition, the 5′-T•A-3′ terminal pairs fray more than the 5′-A•T-3′ pair (14). To check the validity of this differential effect in molecular crowding condition, we included sequences with both these terminal pairs; two sequences (8a and 8b) with terminal 5′-T•A-3′ base pair and four sequences (7a, 7b, 10 and 12) with terminal 5′-A•T-3′ base pair. The nearest-neighbors in the total set of designed oligonucleotides occur with the following frequencies: dAA/dTT = 12, dAT/dTA = 24, dTA/dAT = 11, dCA/dGT = 28, dGT/dCA = 22, dCT/dGA = 22, dGA/dCT = 38, dCG/dGC = 44, dGC/dCG = 31 and dGG/dCC = 32. The minimum and maximum frequencies of occurrence of nearest-neighbor were observed for dTA/dAT and dCG/dGC with 4.2% and 16.7% of the total frequency, respectively. These extremum values are in proximity to the minimum and maximum frequencies of 4.8% and 14.9% for dTA/dAT and dAA/dTT, respectively, with the total nearest-neighbors set reported by SantaLucia *et al.* for calculating improved nearest-neighbor parameters for DNA duplex formation (14).

The structures of all the sequences were checked by CD spectral studies in both dilute and molecular crowding conditions. The CD spectra of all the designed sequences collected at 4°C showed a positive band in the 267–285 nm region and a negative band in the 247–255 nm region (Supplementary Figure S2), corresponding to the CD spectrum of a typical B-type DNA duplex (46,47). The slight alterations in the peak positions and ellipticities of the sequences in the presence of 40 wt% PEG 200 are attributed to the different stabilities of DNA duplexes in the crowding condition (48). Thus, the CD spectral measurements confirmed the duplex structure of the designed self-complementary DNA sequences.

### Melting behavior of self-complementary DNA duplexes in the crowding condition

To verify the validity of the nearest-neighbor model in the crowding condition, we performed UV melting studies of the designed sequences and compared the melting profiles of the oligonucleotides with identical nearest-neighbors since the model assumes similar thermostabilities for oligonucleotides having identical nearest-neighbors. Supplementary Figure S3 presents the melting behaviors of one of the established sequences, d(ATGAGCTCAT) (7a) in the absence and presence of 40 wt% PEG 200 at the same concentration of 100 μM (14). Each melting curve showed two-state transition and melting temperature (*T*_m_) decreased from 46.4°C in the dilute solution to 34.3°C in the crowding condition. The observation is consistent with previous reports, justifying destabilization of Watson–Crick base pair under the molecular crowding condition with PEG 200 (36,38). It is pertinent to mention that we observed a difference (3.9°C) in the experimental and predicted values of *T*_m_ for sequence 7a in the absence of cosolute. This type of differences between experimentally obtained *T*_m_s and their predicted values are frequently observed in a dilute solution, and the difference is found to be more for oligonucleotides having A•T terminal pairs (12,14).

**Figure 1.**
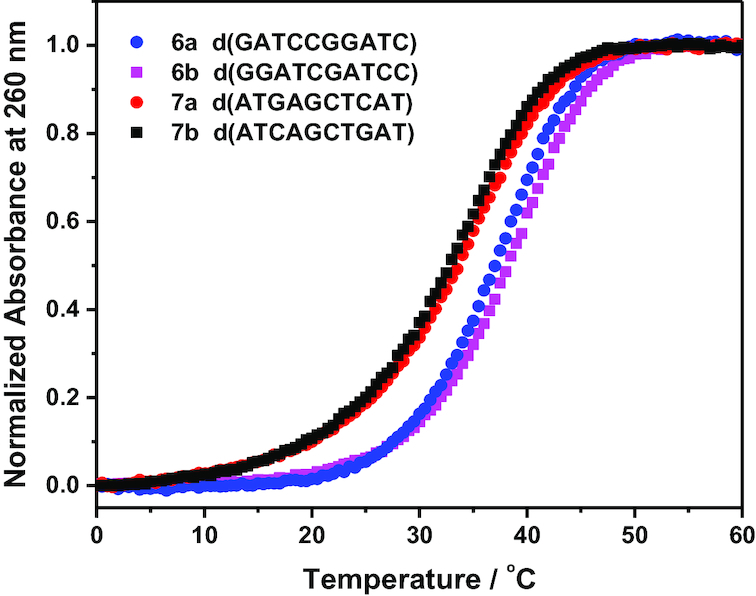
Normalized UV melting curves of d(GATCCGGATC) (6a), d(GGATCGATCC) (6b), d(ATGAGCTCAT) (7a) and d(ATCAGCTGAT) (7b) in buffer containing 0.1 M NaCl, 10 mM Na_2_HPO_4_ (pH 7.0) and 1 mM Na_2_EDTA in the presence of 40 wt% PEG 200. Oligonucleotide sequences are mentioned in the legends. The concentration of these oligonucleotides was 100 μM.

Thereafter, we compared the UV melting behaviors of the designed oligonucleotides having identical nearest-neighbors in the crowding condition. Figure 1 shows UV melting curves of d(GATCCGGATC) (6a) and d(GGATCGATCC) (6b) with G•C pair at each end, and d(ATGAGCTCAT) (7a) and d(ATCAGCTGAT) (7b) with A•T pair at each end in the presence of 40 wt% PEG 200. The melting curves for the sequences in pairs 6 and 7 are almost identical. *T*_m_s were 37.3 and 38.2°C for 6a and 6b, respectively, and 34.3 and 34.0°C for 7a and 7b, respectively (Table 2). These results suggest that duplexes with identical nearest-neighbors possess similar thermostabilities in the crowding environment. This observation is in accordance with our previous study where similar stabilities were observed for sequences d(GTAATTAC) and d(GTTATAAC) and also two other sequences d(GCGGCCGC) and d(GCCGCGGC) containing same nearest-neighbors in the presence of PEG 200 (39). We also measured the UV melting curves for the remaining oligonucleotides in the presence of 40 wt% PEG 200 (figures not shown) and determined *T*_m_s (Table 2). All the self-complementary sequences followed the two-state transition in molecular crowding condition induced by 40 wt% PEG 200, and the melting temperatures were reduced in the crowding condition, compared to the predicted values in dilute solutions (Table 2). Data in Table 2 show that oligonucleotides having identical nearest neighbors (sequence pairs 1–9) exhibit almost similar values of *T*_m_s. Thus, the results validate the nearest-neighbor model in crowding condition for self-complementary DNA duplexes by exhibiting similar degrees of thermostabilities for the oligonucleotides with identical nearest-neighbors.

**Table 2.** Thermodynamic parameters for self-complementary DNA sequences measured in the crowding condition and predicted in the dilute solution

		Measured in the presence of 40 wt% PEG 200^a^				Predicted in the absence of cosolute^b^		Difference in crowding and dilute condition	
No.	Sequence	Δ*H*° (kcal mol^−1^)	*T*Δ*S*° (kcal mol^−1^)	Δ*G*°_37_ (kcal mol^−1^)	*T*_m_^c^ (°C)	Δ*G*°_37_ (kcal mol^−1^)	*T*_m_^c^ (°C)	ΔΔ*G*°_37_ (kcal mol^−1^)	Δ*T*_m_ (°C)
1a	d(CCGCGG)	−49.8 ± 3.4	−44.1 ± 3.1	−5.7 ± 0.5	36.0	−6.6	43.0	0.9	−7.0
1b	d(CGGCCG)	−43.0 ± 3.1	−37.3 ± 2.7	−5.7 ± 0.5	36.9	−6.6	43.0	0.9	−6.1
2a	d(GGACGTCC)	−63.0 ± 2.8	−57.7 ± 2.6	−5.3 ± 0.4	35.4	−7.5	43.1	2.2	−7.7
2b	d(GACCGGTC)	−64.1 ± 6.0	−58.7 ± 5.5	−5.4 ± 0.8	35.6	−7.5	43.1	2.1	−7.5
3a	d(CGTCGACG)	−63.9 ± 2.2	−57.8 ± 2.0	−6.1 ± 0.3	39.0	−7.9	47.6	1.8	−8.6
3b	d(CGACGTCG)	−63.2 ± 3.7	−57.0 ± 3.4	−6.2 ± 0.5	39.3	−7.9	47.6	1.7	−8.3
4a	d(CAAGCTTG)	−79.7 ± 2.4	−76.1 ± 2.3	−3.6 ± 0.3	28.9	−6.2	35.2	2.6	−6.3
4b	d(CTTGCAAG)	−72.5 ± 3.4	−68.6 ± 3.2	−3.9 ± 0.4	29.6	−6.2	35.2	2.3	−5.6
5a	d(CGGTACCG)	−69.5 ± 2.7	−64.9 ± 2.6	−4.6 ± 0.3	32.3	−7.6	40.8	3.0	−8.5
5b	d(CCGTACGG)	−60.5 ± 5.4	−55.1 ± 5.0	−5.4 ± 0.7	34.7	−7.6	40.8	2.2	−6.1
6a	d(GATCCGGATC)	−82.5 ± 5.1	−76.8 ± 4.8	−5.7 ± 0.7	37.3	−8.4	48.7	2.7	−11.4
6b	d(GGATCGATCC)	−77.9 ± 2.2	−71.9 ± 2.0	−6.0 ± 0.3	38.2	−8.4	48.7	2.4	−10.5
7a	d(ATGAGCTCAT)	−71.8 ± 0.9	−66.8 ± 0.8	−5.0 ± 0.1	34.3	−7.6	42.5	2.6	−8.1
7b	d(ATCAGCTGAT)	−77.1 ± 2.0	−72.2 ± 1.9	−4.9 ± 0.3	34.0	−7.6	42.5	2.7	−8.5
8a	d(TGCCGCGGCA)	−71.0 ± 4.7	−61.6 ± 4.1	−9.4 ± 0.8	53.9	−10.3	59.1	0.9	−5.2
8b	d(TGGCGCGCCA)	−61.8 ± 2.7	−54.5 ± 2.6	−9.3 ± 0.1	54.8	−10.3	59.1	1.0	−4.3
9a	d(CATAGGCCTATG)	−86.0 ± 4.3	−79.5 ± 4.0	−6.5 ± 0.6	39.8	−8.8	48.5	2.3	−8.7
9b	d(CTATGGCCATAG)	−91.9 ± 3.6	−85.1 ± 3.4	−6.8 ± 0.5	40.5	−8.8	48.5	2.0	−8.0
10	d(AGTCATGACT)	−69.8 ± 4.3	−65.3 ± 4.0	−4.5 ± 0.5	32.3	−6.9	38.2	2.4	−5.9
11	d(GCGAATTCGC)	−66.9 ± 1.9	−59.9 ± 1.7	−7.0 ± 0.3	43.1	−9.4	51.6	2.4	−8.5
12	d(ATCGCTAGCGAT)	−59.8 ± 9.0	−53.1 ± 8.0	−6.7 ± 1.3	43.1	−9.8	50.3	3.1	−7.2
13	d(GACGACGTCGTC)	−95.0 ± 4.4	−85.9 ± 4.0	−9.1 ± 0.6	48.2	−11.5	59.4	2.4	−11.2
14	d(GCAAGCCGGCTTGC)	−96.2 ± 8.2	−84.0 ± 7.9	−12.2 ± 0.4	58.5	−14.1	67.3	1.9	−8.8
15	d(CGATCGGCCGATCG)	−90.9 ± 8.5	−79.4 ± 8.3	−11.5 ± 0.3	56.9	−13.8	57.3	2.3	−0.4
16	d(CATATGGCCATATG)	−129.4 ± 5.8	−122.2 ± 5.5	−7.2 ± 0.7	40.7	−10.0	53.0	2.8	−12.3
17	d(CAAGATCGATCTTG)	−116.4 ± 9.9	−108.2 ± 9.3	−8.2 ± 1.3	44.6	−10.7	52.8	2.5	−8.2
18	d(CGCGTACGCGTACGCG)	−126.9 ± 6.7	−113.3 ± 6.0	−13.6 ± 1.0	57.9	−16.9	69.8	3.3	−11.9
19	d(CGCAAGCCGGCTTGCG)	−109.4 ± 6.8	−94.6 ± 6.4	−14.8 ± 0.4	64.2	−16.7	72.2	1.9	−8.0

^a^All experiments were performed in a buffer containing 0.1 M NaCl, 10 mM Na_2_HPO_4_ (pH 7.0) and 1 mM Na_2_EDTA.

^b^The values in 0.1 M NaCl were calculated from the values in 1 M NaCl using (equations 3 and 4) as proposed by our group (42). Values in 1 M NaCl were predicted by using the parameters reported by SantaLucia *et al.* (14).

^c^Melting temperatures were calculated for total strand concentration of 100 μM.

### Thermodynamic parameters of duplexes with identical nearest-neighbors in the crowding condition

**Figure 2.**
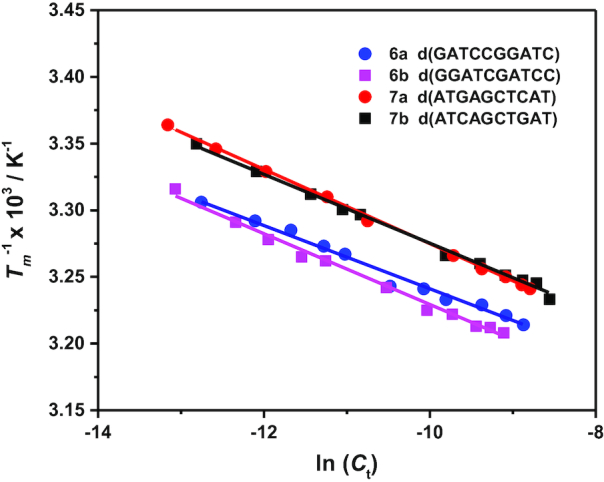
*T*_m_^−1^ versus ln (*C*_t_) plots of d(GATCCGGATC) (6a), d(GGATCGATCC) (6b), d(ATGAGCTCAT) (7a) and d(ATCAGCTGAT) (7b).

Next, the thermodynamic parameters (Δ*H*°, Δ*S*° and Δ*G*°_37_) for the designed sequences were determined from the *T*_m_^−1^ versus ln (*C*_t_) plots in the molecular crowding environment. We have compared the *T*_m_^−1^ versus ln (*C*_t_) plots for the pairs 6 and 7 in Figure 2. Like the melting curves (Figure 1), Figure 2 reveals a similar *T*_m_^−1^ versus ln (*C*_t_) plots for each pair. Thermodynamic parameters for 6a and 6b were −82.5 and −77.9 kcal mol^−1^ for Δ*H*°, −76.8 and −71.9 kcal mol^−1^ for *T*Δ*S*° and −5.7 and −6.0 kcal mol^−1^ for Δ*G*°_37_. Those for 7a and 7b were −71.8 and −77.1 kcal mol^−1^ for Δ*H*°, −66.8 and −72.2 kcal mol^−1^ for *T*Δ*S*°, and −5.0 and −4.9 kcal mol^−1^ for Δ*G*°_37_. We calculated the thermodynamic parameters for all the designed sequences in the crowding condition and the values are summarized in Table 2. Table 2 reflects that the oligonucleotides in pairs 1–9 have similar thermodynamic parameters and *T*_m_. The average differences in the percentage of Δ*H*°, *T*Δ*S*° and Δ*G*°_37_, for sequence in pairs 1–9 are 9.6%, 8.4% and 4.3%, respectively, that might be ascribed to the experimental errors. The average difference in *T*_m_ (0.8°C) was also small. Similar magnitudes of differences (7.7%, 8.2%, 6.5% and 2.3°C for Δ*H*°, *T*Δ*S*°, Δ*G*°_37_ and *T*_m_, respectively) were also observed for pairs of RNA/DNA hybrid sequences with identical nearest-neighbors in the absence of cosolute (14). These results clearly suggest that the nearest-neighbor model is valid even in the molecular crowding condition, since the model predicts that the pairs with identical nearest neighbors will have identical thermostability and thermodynamic parameters for duplex formation.

### Thermodynamic parameters of oligonucleotides having identical nearest-neighbors in the crowding condition induced by different cosolutes

Intracellular environment is crowded by different types of biomolecules, from small amino acids to large protein molecules, differing largely in molecular weights (29). Therefore, it is reasonable to cross-check the validity of the nearest-neighbor model in the crowding condition induced by cosolutes other than PEG 200, which differ largely in their molecular structure. We chose dextran 70 and Ficoll 70 as cosolutes, both having a molecular weight of 70 000, and oligonucleotides pairs 6 and 7 having different initiation factor. Although the solubility of these cosolutes allowed the preparation of 20 wt% solutions, it is known that the stability of the duplex structure is linearly related to the concentration of the cosolutes (37,38). Experimental results are summarized in Table 3. Data in Table 3 show that in the presence of larger cosolutes, sequences having identical nearest-neighbors exhibit almost identical thermodynamic parameters and *T*_m_s. It is pertinent to mention that, unlike PEG 200, in the case of dextran and Ficoll, oligonucleotides were slightly stabilized in the crowding conditions (refer Table 2 for predicted values of Δ*G*°_37_ and *T*_m_s in the absence of cosolute). This observation is in agreement with the previous report of stabilization of duplex DNA structure by high molecular weight cosolutes (32,36,49). Since the stability of the oligonucleotides directly depends on the nature of cosolutes, differences in the stabilities of these sequences, in the solutions of dextran and Ficoll, are due to the different nature of these cosolutes (36). Almost identical thermodynamic parameters, for sequences with identical nearest-neighbors in the presence of different cosolutes, imply that the validity of the model is independent of the nature of cosolutes.

**Table 3.** Thermodynamic parameters for sequences with identical nearest-neighbors measured in the molecular crowding condition induced by dextran 70 and Ficoll 70^a^

Condition	Sequences	Δ*H*° (kcal mol^−1^)	*T*Δ*S*° (kcal mol^−1^)	Δ*G*°_37_ (kcal mol^−1^)	*T*_m_ (°C)
20 wt% dextran 70	d(GATCCGGATC) (6a)	−72.0 ± 3.3	−63.4 ± 3.2	−8.6 ± 0.15	49.0
	d(GGATCGATCC) (6b)	−73.0 ± 2.0	−64.3 ± 1.9	−8.7 ± 0.10	49.1
	d(ATGAGCTCAT) (7a)	−66.9 ± 2.3	−59.0 ± 2.2	−7.9 ± 0.10	45.1
	d(ATCAGCTGAT) (7b)	−68.5 ± 2.5	−60.8 ± 2.3	−7.7 ± 0.13	45.2
20 wt% Ficoll 70	d(GATCCGGATC) (6a)	−78.0 ± 1.8	−69.0 ± 1.7	−9.0 ± 0.10	51.5
	d(GGATCGATCC) (6b)	−79.6 ± 2.2	−70.4 ± 2.0	−9.2 ± 0.14	51.0
	d(ATGAGCTCAT) (7a)	−66.4 ± 3.0	−58.5 ± 2.9	−7.9 ± 0.12	46.1
	d(ATCAGCTGAT) (7b)	−68.2 ± 2.3	−60.2 ± 3.2	−8.0 ± 0.11	46.5

^a^All the experiments were performed in buffer containing 0.1 M NaCl, 10 mM Na_2_HPO_4_ (pH 7.0) and 1 mM Na_2_EDTA in the presence of 20 wt% cosolutes. Melting temperatures were calculated for total strand concentration of 100 μM.

### Relationship between stabilities of DNA duplexes measured in the crowding condition and those predicted with the nearest-neighbor model in the dilute condition

We investigated the quantitative relationship between the stabilities of DNA duplexes measured in the crowding condition induced by 40 wt% PEG 200 and those predicted with the nearest-neighbor model in the absence of cosolute. The stabilities of the designed sequences are predicted with the parameters reported by SantaLucia *et al.* (14). However, the parameters reported therein were applicable for solutions containing 1 M NaCl. Since, in this work, we have measured all the thermodynamic parameters in physiological salt concentration, i.e., 0.1 M NaCl, we estimated Δ*G*°_37_ as well as *T*_m_ values for the oligonucleotides in 0.1 M NaCl, from their values in 1 M NaCl using the following equations as proposed by our group (42).

(3) \begin{equation*}\Delta G{^\circ _{37}}\left( {0.1\,{\rm{M}}} \right) = 0.63\,\Delta G{^\circ _{37}}\left( {1\,{\rm{M}}} \right)-1.67\end{equation*}

(4) \begin{equation*}{T_{\rm m}}\left( {0.1\,{\rm{M}}} \right) = 0.88{T_{\rm m}}\left( {1\,{\rm{M}}} \right)-5.15\end{equation*}

**Figure 3.**
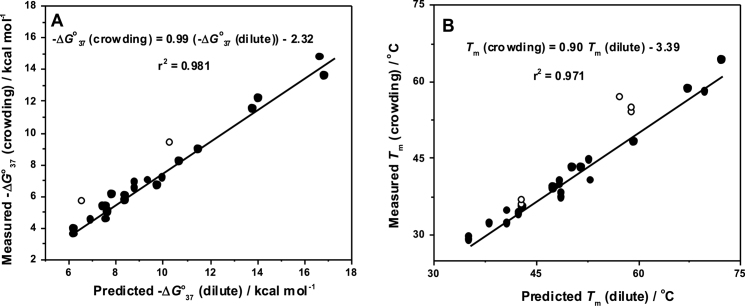
Relationship between the measured (**A**) Δ*G*°_37_ and (**B**) *T*_m_ in the molecular crowding condition and those predicted in the absence of cosolute by the nearest-neighbor parameters. Least-square fits are shown as straight lines, and *r*^2^ represents their correlation coefficients. White circles correspond to the sequences of pairs 1 and 8. In (B), the additional white circle corresponds to the *T*_m_ of oligonucleotide d(CGATCGGCCGATCG). White circles are not included in the fitted lines.

The predicted values of Δ*G*°_37_ and *T*_m_ for all the designed sequences in 0.1 M NaCl in the absence of cosolute are presented in Table 2 along with the measured values in the presence of 40 wt% PEG 200. We employed a linear relationship between Δ*G*°_37_ and *T*_m_ values measured in the crowding condition and those predicted in the absence of cosolute using the nearest-neighbor model as depicted in Figure 3. The fitted straight lines in Figure 3 produced the following equations:

(5) \begin{equation*}\Delta G{^\circ _{37}}\left( {{\rm{crowding}}} \right) = 0.99\,\Delta G{^\circ _{37}}\left( {{\rm{dilute}}} \right) + 2.32\end{equation*}

(6) \begin{equation*}{T_{\rm m}}\left( {{\rm{crowding}}} \right) = 0.90{T_{\rm m}}\left( {{\rm{dilute}}} \right)-3.39\end{equation*}

For the fitting of straight lines in Figure 3, we did not incorporate the data points corresponding to pairs 1 and 8 as they fall far apart. Equations (5) and (6) indicate that Δ*G*°_37_ and *T*_m_ values for self-complementary DNA sequences in the crowding condition can be easily calculated by using the existing nearest-neighbor parameters. The predicted values agreed well with the measured Δ*G*°_37_ and *T*_m_ values in the crowding condition (Table 4) with an average difference of 4.7% and 1.2°C, respectively, which is a quite decent estimation compared to the average difference of 5.7% and 2.4°C, respectively, for the prediction of Δ*G*°_37_ and *T*_m_ values in 0.1 M NaCl from their values in 1 M NaCl by using similar linear relations (equations 3 and 4) (42). Since we did not determine the nearest-neighbor parameters in the crowding condition, the goodness of the prediction systems (equations 5 and 6) will depend on the existing nearest-neighbor parameters used here to predict stabilities in the dilute condition and their inherent assumptions. However, to assess the predictive nature of equations (5) and (6), we employed leave-one-out analysis in which one test sequence was chosen and kept out of the fitting in Figure 3 to obtain the linear relation by fitting the remaining sequences. The resulting equations were used to predict the Δ*G*°_37_ and *T*_m_ values for the test sequence, and the same procedure was repeated for every sequence in the fitted lines. We obtained an average difference of 4.6% and 1.1°C, respectively, for Δ*G*°_37_ and *T*_m_ between the predicted and experimental values, suggesting the goodness of our prediction systems (equations 5 and 6).

**Table 4.** Comparison between measured and predicted values of Δ*G*°_37_ and *T*_m_ along with the calculated Δ*G*°_37_ values in the crowding condition (40 wt% PEG200)

		Measured		Predicted		Calculated
No.	Sequence	Δ*G*°_37_ (kcal mol^−1^)	*T*_m_ (°C)	Δ*G*°_37_ (kcal mol^−1^)	*T*_m_ (°C)	Δ*G*°_37_ (kcal mol^−1^)
1a	d(CCGCGG)	−5.7	36.0	−4.2	35.3	−4.7^a^
1b	d(CGGCCG)	−5.7	36.9	−4.2	35.3	
2a	d(GGACGTCC)	−5.3	35.4	−5.1	35.4	−5.5^a^
2b	d(GACCGGTC)	−5.4	35.6	−5.1	35.4	
3a	d(CGTCGACG)	−6.1	39.0	−5.5	39.5	−5.9^a^
3b	d(CGACGTCG)	−6.2	39.3	−5.5	39.5	
4a	d(CAAGCTTG)	−3.6	28.9	−3.8	28.3	−4.1^a^
4b	d(CTTGCAAG)	−3.9	29.6	−3.8	28.3	
5a	d(CGGTACCG)	−4.6	32.3	−5.2	33.3	−5.3^a^
5b	d(CCGTACGG)	−5.4	34.7	−5.2	33.3	
6a	d(GATCCGGATC)	−5.7	37.3	−6.0	40.4	−6.2^a^
6b	d(GGATCGATCC)	−6.0	38.2	−6.0	40.4	
7a	d(ATGAGCTCAT)	−5.0	34.3	−5.2	34.9	−4.7^a^
7b	d(ATCAGCTGAT)	−4.9	34.0	−5.2	34.9	
8a	d(TGCCGCGGCA)	−9.4	53.9	−7.9	49.8	−8.5^a^
8b	d(TGGCGCGCCA)	−9.3	54.8	−7.9	49.8	
9a	d(CATAGGCCTATG)	−6.5	39.8	−6.4	40.3	−6.4^a^
9b	d(CTATGGCCATAG)	−6.8	40.5	−6.4	40.3	
10	d(AGTCATGACT)	−4.5	32.3	−4.5	31.0	−4.7
11	d(GCGAATTCGC)	−7.0	43.1	−7.0	43.1	−7.2
12	d(ATCGCTAGCGAT)	−6.7	43.1	−7.4	41.9	−7.4
13	d(GACGACGTCGTC)	−9.1	48.2	−9.1	50.1	−9.3
14	d(GCAAGCCGGCTTGC)	−12.2	58.5	−11.6	57.2	−11.7
15	d(CGATCGGCCGATCG)	−11.5	56.9	−11.3	48.2	−11.5
16	d(CATATGGCCATATG)	−7.2	40.7	−7.6	44.3	−7.4
17	d(CAAGATCGATCTTG)	−8.2	44.6	−8.3	44.1	−8.1
18	d(CGCGTACGCGTACGCG)	−13.6	57.9	−14.4	59.4	−14.4
19	d(CGCAAGCCGGCTTGCG)	−14.8	64.2	−14.2	61.6	−14.3

^a^For the numerical calculation, the measured Δ*G*°_37_ of the sequence of a and b were averaged.

### Calculation of the nearest-neighbor parameters of self-complementary sequences in the crowding condition

**Figure 4.**
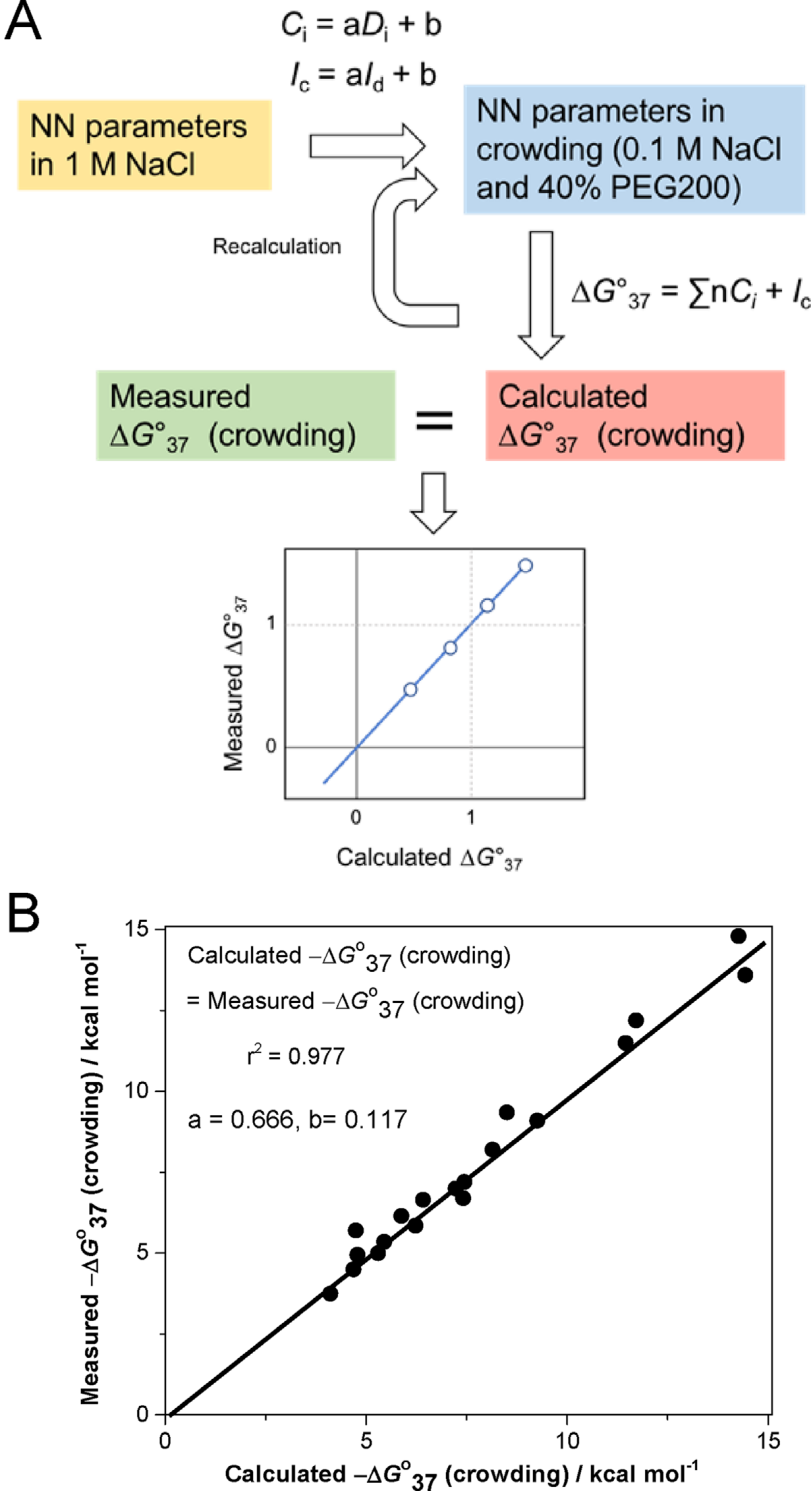
Calculation by numeric approach of Δ*G*°_37_ (crowding) to determine the nearest-neighbor parameters in the crowding condition from established nearest-neighbor parameters. (**A**) Schematic illustration of the analysis and (**B**) the best found correlation between the calculated and measured Δ*G*°_37_ (crowding).

Since the stability of the oligonucleotide can be the sum of individual nearest-neighbor (NN) stabilities, equation (5) indicated that the NN parameters in the crowding conditions can be calculated by a simple linear approximation from the parameters determined in a dilute condition; it means that Δ*G*°_37_ from the NN parameters (crowding) can be shown by Δ*G*°_37_ from the NN parameters (dilute) as follows:

(7) \begin{equation*}\Delta G{^\circ _{37}}\,{\rm{(crowding)}} = \sum\limits_i {{{n}}\,{C_{i}} + {I_{\rm c}}} \end{equation*}

(8) \begin{equation*}{C_i} = {{a}}{D_i}\ + {{b}}\end{equation*}

(9) \begin{equation*}{I_{\rm c}} = {{a}}{I_{\rm d}}\ + {{b}}\end{equation*}

where *C* is Δ*G*°_37_ from the NN parameters in crowding, *i* is each case of the 10 NN base sets, *n* is the number of frequencies of the NN parameters of the NN set *i* in each sequence (Table 1), *I*_c_ is the initiation factor in the crowding condition, *D* is Δ*G*°_37_ from the NN parameters in dilute solution containing 1 M NaCl established by SantaLucia *et al.* (14), and *I*_d_ is the initiation factor in the same dilute condition. In addition, *a* and *b* are the constant values. Here, we omitted the symmetry factor, because the self-complementary sequences were only used in this study. First, we wrote 19 equations of measured NN sets in this study using equation (7). Then, we numerically calculated Δ*G*°_37_ (crowding) by varying the value of *a* and *b*. Next, we compared the obtained Δ*G*°_37_ (crowding) with experimentally obtained Δ*G*°_37_ in the crowding conditions (Table 2). To confirm the correlation between calculated Δ*G*°_37_ (crowding) and measured Δ*G*°_37_ (crowding), we plotted each value and analyzed by linear regression (Figure 4A). If the calculated and measured Δ*G*°_37_ perfectly match each other, we can get a linear relationship where the slope is 1 and intercept is 0. To obtain the best answer for equation (7), we further calculated various combinations of both *a* and *b* values to make a perfect match of the calculated and measured Δ*G*°_37_, and found that *a* = 0.666 and *b* = 0.117, where the measured Δ*G*°_37_ (crowding) versus the calculated Δ*G*°_37_ (crowding) plots displayed good linear correlation (correlation coefficient (*r*^2^) = 0.977) with the slope of almost 1 (1.0006) and the intercept of almost 0 (−0.0004) (Figure 4B). Therefore, we conclude the NN parameters in the crowding condition can be determined as follows:

(10) \begin{eqnarray*}\Delta G{^\circ _{37}}\left( {{\rm{crowding}}} \right) &=& 0.666\,\Delta G{^\circ _{37}}\left( {{\rm{dilute}},\,1\,{\rm{M}}\,{\rm{NaCl}}} \right)\nonumber\\ && + 0.117\end{eqnarray*}

All the NN parameters are calculated by equation (10) and shown in Table 5. Errors in the NN parameters in crowding condition were calculated from the reported errors for those parameters in dilute solution by SantaLucia *et al.* (14), following the standard procedure of error analysis. The calculated Δ*G°*_37_ values of each sequence are shown in Table 4.

**Table 5.** Calculated nearest-neighbor parameters for Δ*G*°_37_ in the presence of 0.1 M NaCl and 40 wt% PEG200

Nearest-neighbor set	Calculated Δ*G*°_37_ (kcal mol^−1^)
dAA/dTT	−0.56 ± 0.03
dAT/dTA	−0.37 ± 0.03
dTA/dAT	−0.28 ± 0.03
dCA/dGT	−0.80 ± 0.04
dGT/dCA	−0.84 ± 0.03
dCT/dGA	−0.66 ± 0.05
dGA/dCT	−0.86 ± 0.03
dCG/dGC	−1.27 ± 0.05
dGC/dCG	−1.40 ± 0.05
dGG/dCC	−1.06 ± 0.04
Initiation GC	1.33 ± 0.16
Initiation AT	1.98 ± 0.67

## DISCUSSION

In the present study, we verified the applicability of the nearest-neighbor model for self-complementary DNA duplexes in the molecular crowding condition induced by 40 wt% PEG 200. The similarity in the thermodynamic parameters (Δ*H*°, *T*Δ*S*° and Δ*G*°_37_) and melting temperatures (*T*_m_s) for different pairs of oligonucleotides with identical nearest-neighbors confirmed the validity of the model in the molecular crowding conditions. Stability of the DNA duplexes in the crowding condition is mainly explained by water activity and excluded volume effect (38,40). Cosolutes with low molecular weights, such as ethylene glycol, 1,3-propanediol, 1,2-dimethoxyethane and PEG 200, reduce the stability of the duplexes due to the lowered water activity (36,38). On the other hand, large cosolutes like PEG 8000, dextran and Ficoll enhance the duplex stability through the excluded volume effect (32,49). Our experimental results also followed the same trend. The validity of the model in the crowding condition suggested that all the parameters depending on 10 nearest-neighbor pairs should be affected in a similar manner, although to different extents. To have precise quantification about the effect of crowding condition on the nearest-neighbor parameters, we will have to evaluate the thermodynamic parameters for the 10 nearest-neighbors in the molecular crowding condition. The nearest-neighbor model predicts nucleic acid stabilities by considering the major interactions in a nucleic acid duplex formation, i.e., stacking interaction between nearest-neighbor bases and hydrogen bonding interaction in a base pair. As these interactions are conserved to different extents in crowding conditions, the model also remains valid in the crowding condition. Similar thermodynamic values for the pairs of oligonucleotides (pairs 1–9) having nucleotide chain length of 6 to 12 in the crowding condition (Table 2) indicate that validity of the model in the crowding condition does not depend on the length of the oligonucleotides, at least for shorter duplexes. Our results also suggest that the model is valid even in the presence of dextran and Ficoll having large differences in molecular weights from PEG 200.

A linear relationship existed between the measured values and those predicted by the nearest-neighbor model (equations 5 and 6) in dilute solution. However, we found anomalies for some oligonucleotide sequences and all the data points were not incorporated in Figure 3. Sequences in pair 1 showed large deviation from the fitted straight line. It was suggested that the G•C base pair is more stable than the A•T pair in a physiological buffer condition (50). Therefore, predicted stabilities for the sequences d(CCGCGG) (1a) and d(CGGCCG) (1b) in the absence of cosolute (Δ*G*°_37_ is −6.6 kcal mol^−1^ for both 1a and 1b, Table 2) were considerably higher compared to the 6-mer oligonucleotides containing both G•C and A•T pairs in dilute condition, as reported earlier (14). These oligonucleotides also exhibited high stability (Δ*G*°_37_ value is −5.7 kcal mol^−1^ for both 1a and 1b, Table 2) in the crowding condition. The relative smaller effect of destabilization than expected may be due to the excluded volume effect by PEG200, because the volumetric change of the formation of G•C base pair is larger than that of A•T pair (51). Thus, the data points of these two sequences deviate from linearity in Figure 3A; however, the thermodynamic parameters for these two oligonucleotides (1a and 1b) in the crowding condition were similar, confirming the validity of the nearest-neighbor model in the crowding condition. Sequences d(TGCCGCGGCA) (8a) and d(TGGCGCGCCA) (8b) also differed from the linear fits for Δ*G*°_37_ (Figure 3A) and *T*_m_ (Figure 3B). It is reported that in the absence of cosolutes terminal fraying of the 5′-T•A-3′ pair enhances the stability of the sequences by a favorable entropy change, and the effect is found to be more prominent in the 5′-T•A-3′ pair compared to the 5′-A•T-3′ pair (52). In the crowding condition, we found relatively higher stabilities for the sequences 8a and 8b, and this was due to similar fraying effect. Therefore, data points corresponding to these sequences deviated from the fitted lines. The Δ*G*°_37_ and *T*_m_ values for sequences d(ATGAGCTCAT) (7a) and d(ATCAGCTGAT) (7b) containing 5′-A•T-3′ terminal base pair fall in the fitted line, suggesting less terminal fraying effect for 5′-A•T-3′ terminal pair in the crowding condition. The *T*_m_ value for oligonucleotide d(CGATCGGCCGATCG) (15) deviated largely from the linear fit (Figure 3B). The high *T*_m_ for the sequence in the crowding condition may be due to favorable entropy change; however, this requires further consideration.

Presently, we used only self-complementary DNA sequences. Thus, the use of equations (5) and (6) should be limited to predict the stabilities of self-complementary DNA duplexes only, in the crowding environments induced by small cosolutes. The stability of the DNA duplex depends on the nature of cosolutes as well as the length of the oligonucleotides (36). Thus, the slope and intercept of the equations for predicting Δ*G*°_37_ and *T*_m_ may depend on the cosolutes and length of the oligonucleotides. In the present study, we did not consider the length of the oligonucleotides for the prediction systems. However, we found a good correlation between the measured and predicted values (correlation coefficients of 0.981 and 0.971 for Δ*G*°_37_ and *T*_m_, respectively). Therefore, equations (5) and (6) can be applied to predict the stabilities of short duplexes in crowding environments with small cosolutes. Corrections in the size of the cosolute and the length of the duplex in these equations will provide a comprehensive prediction of duplex stabilities in various crowding conditions.

Finally, we determined the nearest-neighbor parameters in the crowding conditions by the calculation from 19 measured Δ*G*°_37_ values based on the approximation that Δ*G*°_37_ (crowding) can be converted from the established Δ*G*°_37_ (dilute) using a simple linear relation. The correlation coefficient of the plot of the measured Δ*G*°_37_ (crowding) versus the calculated Δ*G*°_37_ (crowding) was 0.977, suggesting that the nearest-neighbor parameters can be determined as well as those determined in the dilute solution, by a simple treatment such as linear regression. Moreover, the correlation coefficient was almost the same as that of the prediction using equation (5) (0.981), even including the data that were excluded to obtain the equation (5). Therefore, both methods for the prediction of Δ*G*°_37_ values in the crowding conditions are useful and reliable.

In summary, we demonstrated the validity of the nearest-neighbor model for self-complementary DNA sequences in the molecular crowding condition of PEG 200 as well as dextran 70 and Ficoll 70. A simple prediction system has been obtained for the gross prediction of Δ*G*°_37_ and *T*_m_ values in the crowding condition from the values in the dilute solution, calculated by nearest-neighbor parameters. The prediction of stabilities of self-complementary DNA duplexes in cell-like crowded environments will be helpful in techniques like antisense oligonucleotide therapy, DNA origami techniques for developing medical tools, as well as in understanding the role of DNA duplex in fundamental biological processes more explicitly.

## Supplementary Material

Supplementary Data
