# Supplementary material for: Validation of the nearest-neighbor model for Watson–Crick self-complementary DNA duplexes in molecular crowding condition
Source: Nucleic Acids Res. 2019 Feb 8;47(7):3284–94. doi: 10.1093/nar/gkz071 (PMC6468326; doi:10.1093/nar/gkz071)
Supplement: Supplementary Data [file gkz071_supplemental_file.pdf]

Supporting Information

Validation of the Nearest-neighbor Model for Watson–Crick Self-complementary DNA Duplexes in Molecular Crowding Condition

Saptarshi Ghosh,[1] Shuntaro Takahashi,[1] Tamaki Endoh,[1] Hisae Tateishi-Karimata,[1] Soumitra Hazra,[1] and Naoki Sugimoto[1, 2,*]

[1]Frontier Institute for Biomolecular Engineering Research (FIBER), [2]Graduate School of Frontiers of Innovative Research in Science and Technology (FIRST), Konan University, Kobe, Japan

Supporting Method

Determination of T_m from the UV Melting Curve

Self-complementary duplex formation can be represented as follows:

$$2A \rightleftharpoons A_2 \qquad (1)$$

where, A and A_2 indicate the single-stranded and the double-stranded DNA, respectively. If α is the fraction of total strand concentration and C_t, is the concentration of the duplex, then absorbance (A) at temperature (T) can be calculated as:

$$A = C_t\, l\, [\varepsilon_{ss} (1- \alpha) + \varepsilon_{ds}\, \alpha/2] \qquad (2)$$

where, ε_{ss} and ε_{ds} are the extinction coefficients for the single-stranded and double-stranded DNA, respectively, and l represents the length of the cuvette used. Extinction coefficients are temperature dependent and in general, the temperature dependence is assumed to be linear,

$$\varepsilon_{ss} = m_{ss}T + b_{ss} \qquad (3)$$

$$\varepsilon_{ds} = m_{ds}T + b_{ds} \qquad (4)$$

where, m_{ds} and $b_{ds,}$ and m_{ss} and b_{ss} represent the slope and intercept of the lower baseline and upper baseline,respectively, for the melting curve of a duplex dissociation, i.e., where only single strands or only duplexes occur.

To obtain the T_m value from a melting curve, first we drew upper and lower baselines in the linear region of the low and high temperatures (Figure S1) by taking 15 to 20 data points. Then, the median between upper and lower baselines was drawn. The temperature at which the median intersected the melting curve was T_m for the oligonucleotide at any particular concentration.

Supporting Figures

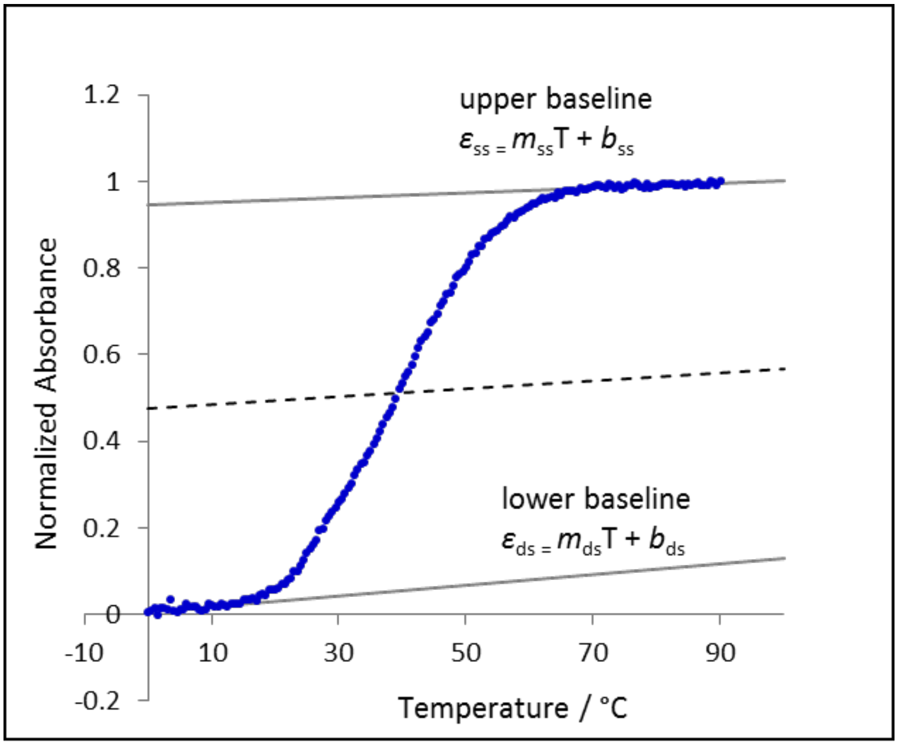

Figure S1. Upper and lower baselines for the UV melting curve. Upper and lower baselines can be represented as $\varepsilon_{ss} = m_{ss}T + b_{ss}$ and $\varepsilon_{ds} = m_{ds}T + b_{ds}$, respectively, where ε_{ss} and ε_{ds} are the extinction coefficients for the single-stranded and double-stranded DNA, respectively. The m_{ds} and b_{ds} or m_{ss} and b_{ss} represent the slope and intercept of the lower base line or upper base line for the melting curve, respectively. The dashed line represents the median of the upper and lower baselines.

(a)

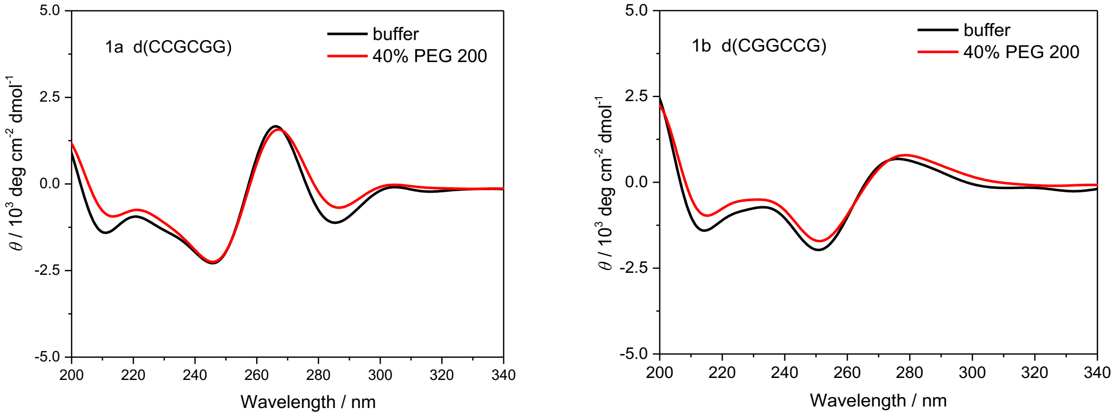

(b)

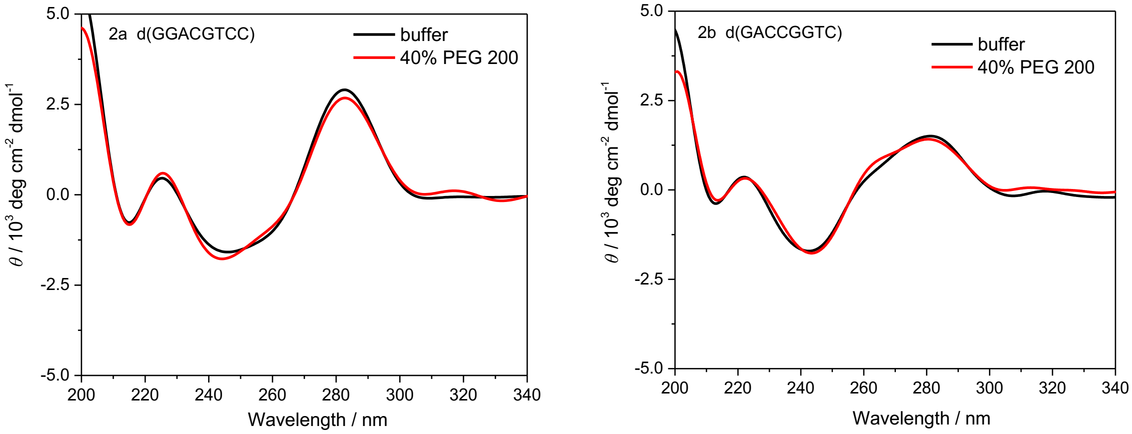

(c)

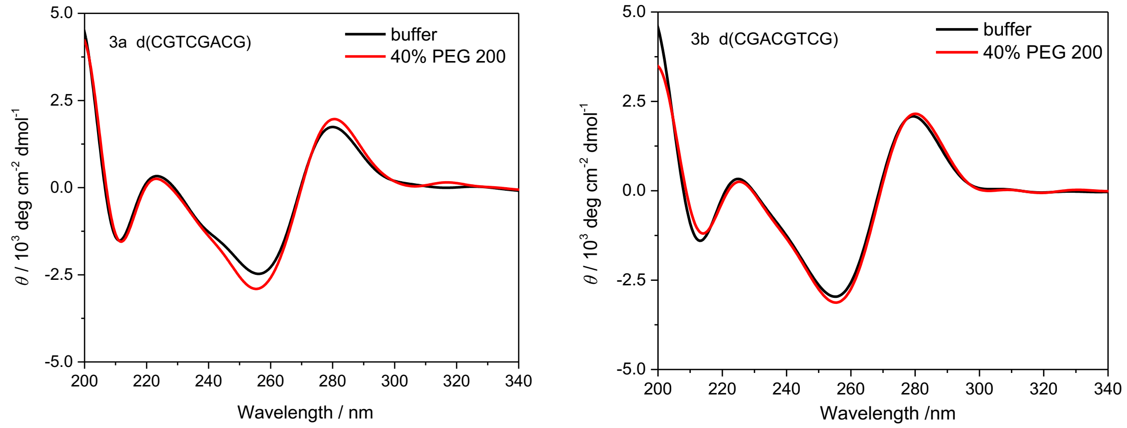

Figure S2 Continued

(d)

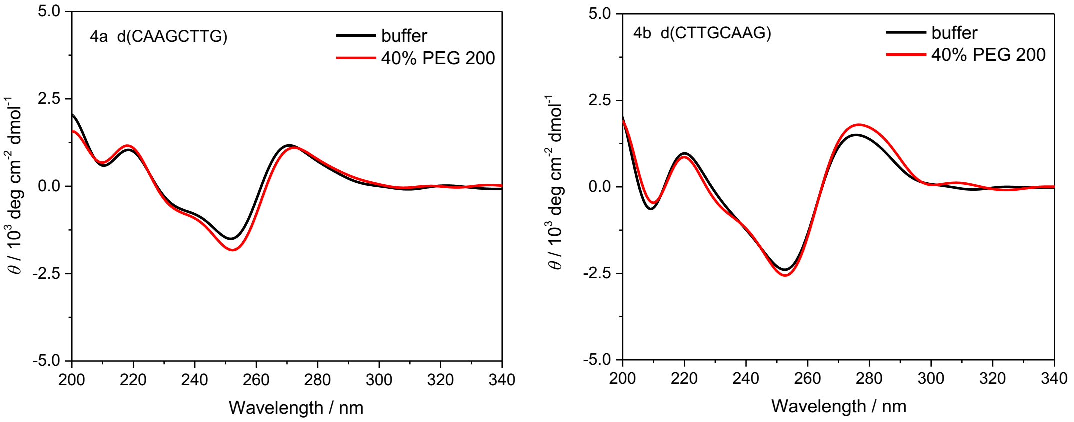

(e)

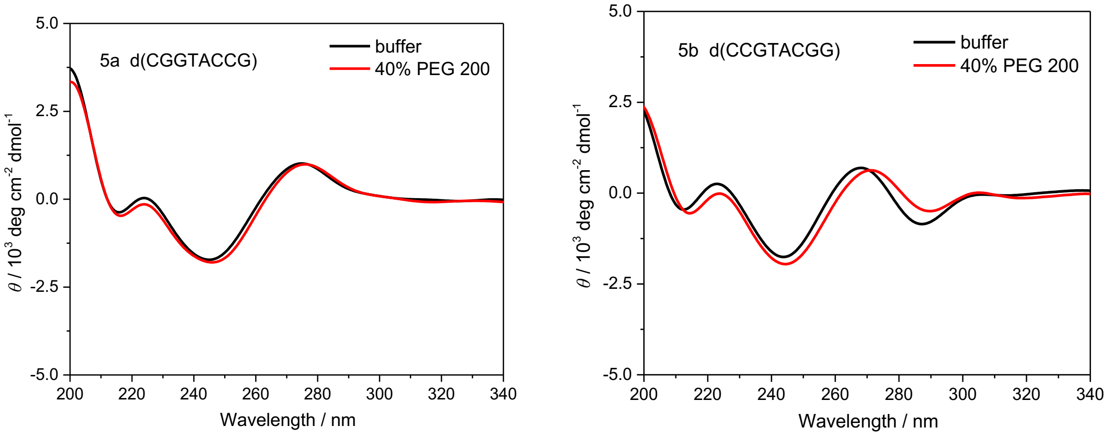

(f)

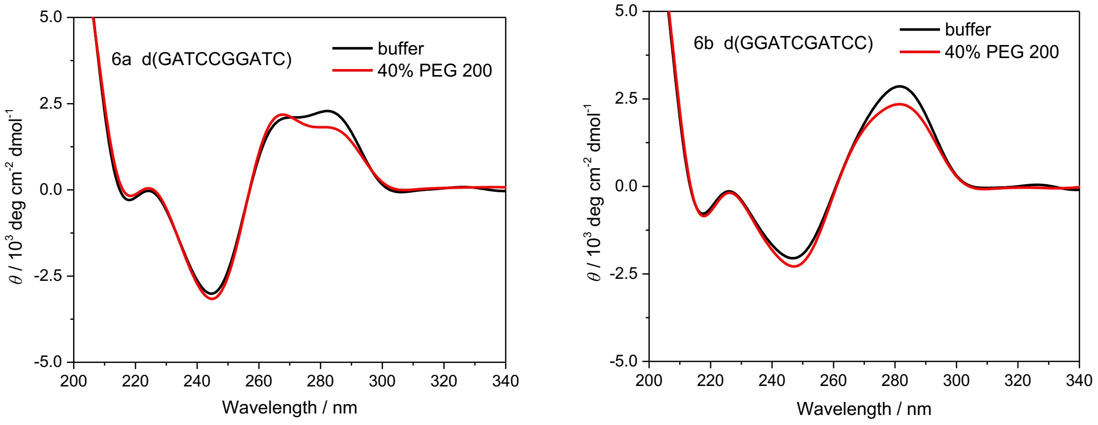

Figure S2 Continued

(g)

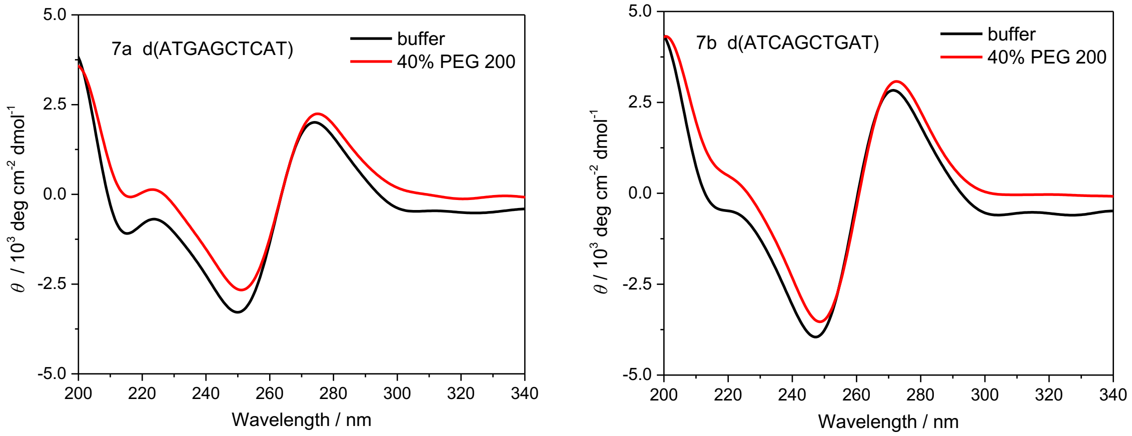

(h)

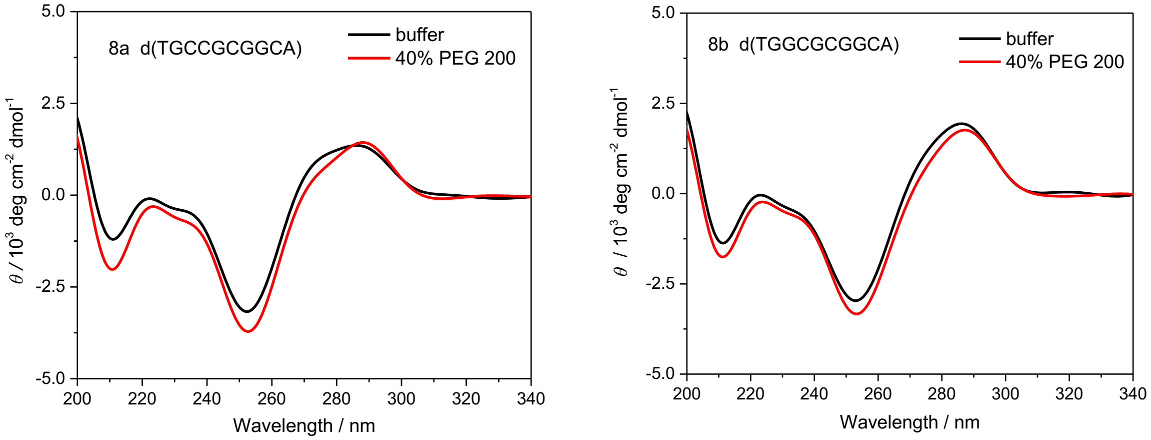

(i)

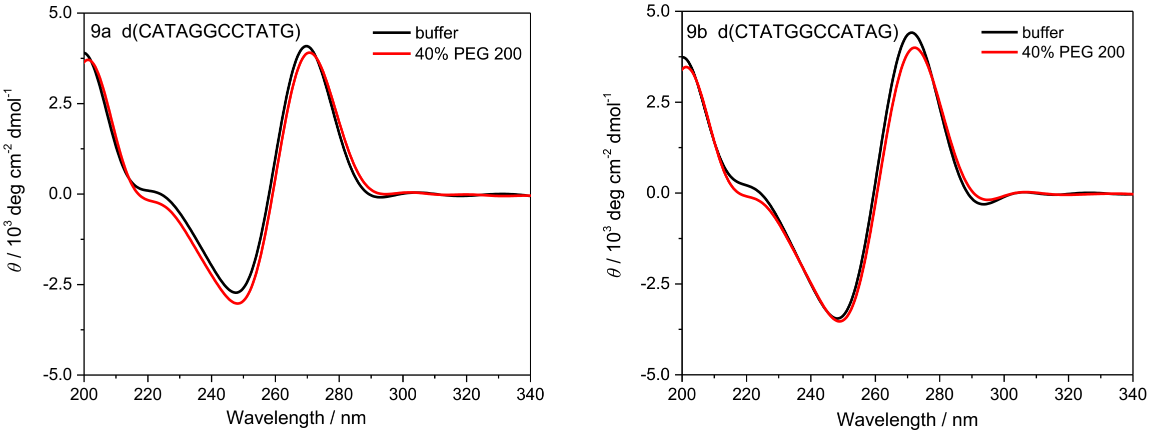

Figure S2 Continued

(j)

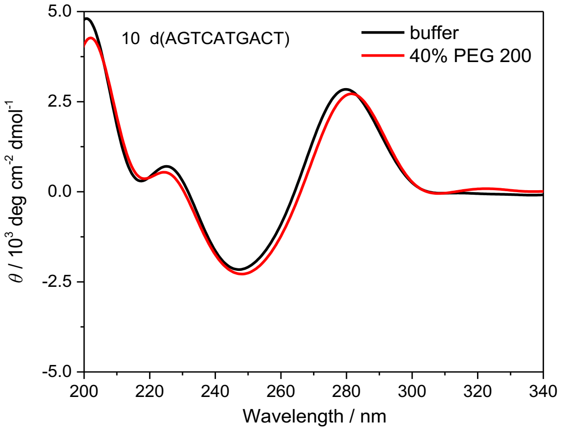

(k)

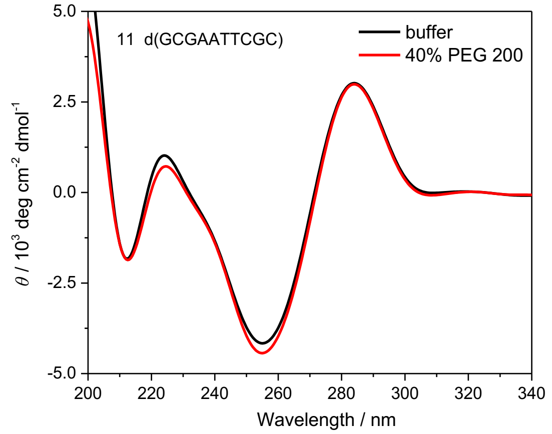

(l)

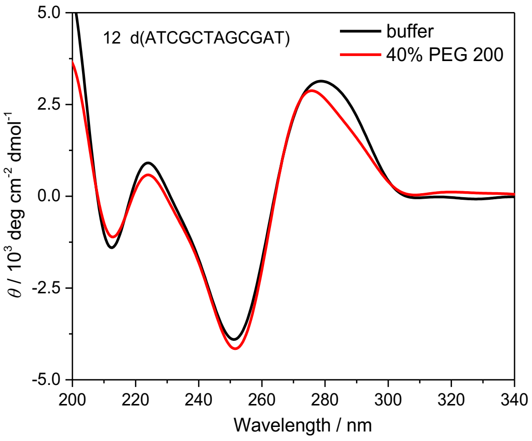

(m)

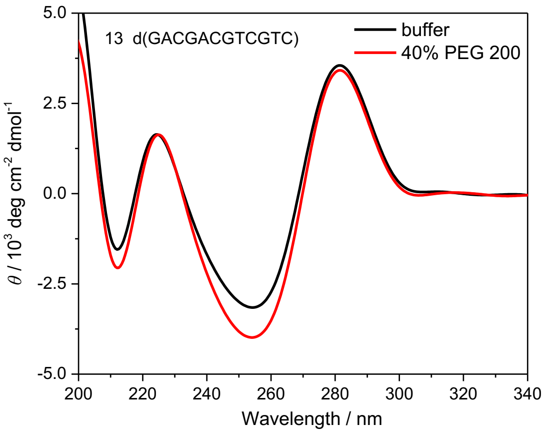

(n)

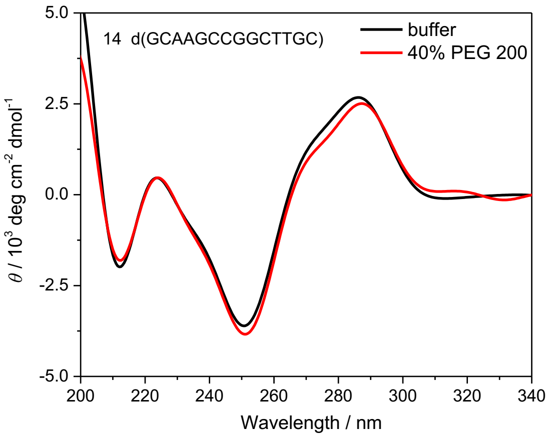

(o)

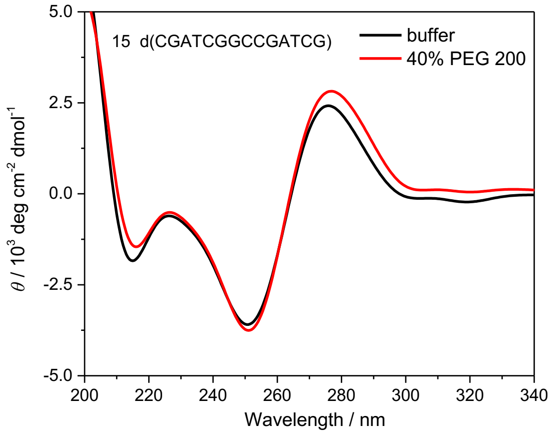

Figure S2 Continued

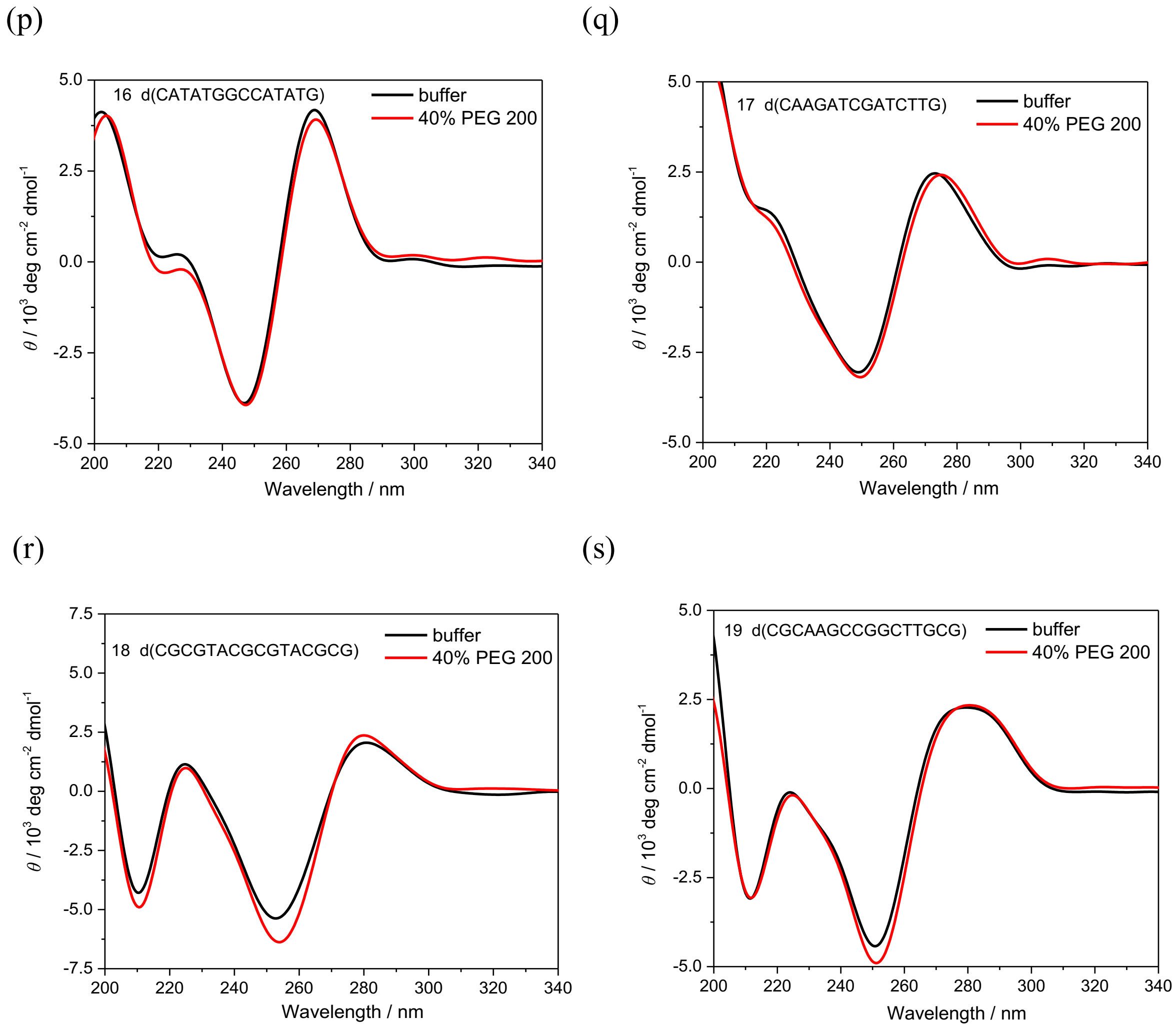

Figure S2. CD spectra of 20 μM oligonucleotides (a) No 1a and 1b, (b) No 2a and 2b, (c) No 3a and 3b, (d) No 4a and 4b, (e) No 5a and 5b, (f) No 6a and 6b, (g) No 7a and 7b, (h) No 8a and 8b, (i) No 9a and 9b, (j) No 10, (k) No 11, (l) No 12, (m) No 13, (n) No 14, (o) No 15, (p) No 16, (q) No 17, (r) No 18 and (s) No 19. All the assays were carried out at 4°C in a buffer containing 0.1 M NaCl, 10 mM Na_2HPO_4 (pH 7.0) and 1 mM Na_2EDTA in the absence (black) and presence (red) of 40 wt% PEG 200. Oligonucleotide sequences are mentioned in the legends.

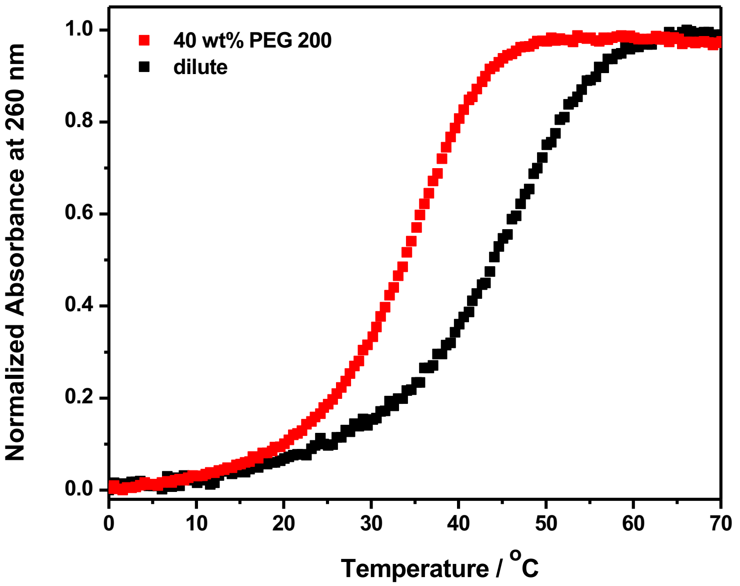

Figure S3. Normalized UV melting curves of d(ATGAGCTCAT) (7a) in 0.1 M NaCl-phosphate buffer in the absence (black) and presence (red) of 40 wt% PEG 200. The concentration of the oligonucleotide was 100 μM.
